# Mixtures of tense and relaxed state polymerized human hemoglobin regulate oxygen affinity and tissue construct oxygenation

**DOI:** 10.1371/journal.pone.0185988

**Published:** 2017-10-11

**Authors:** Donald Andrew Belcher, Uddyalok Banerjee, Christopher Michael Baehr, Kristopher Emil Richardson, Pedro Cabrales, François Berthiaume, Andre Francis Palmer

**Affiliations:** 1 William G. Lowrie Department of Chemical and Biomolecular Engineering, College of Engineering, The Ohio State University, Columbus, Ohio, United States of America; 2 Department of Biomedical Engineering, College of Engineering, The Ohio State University, Columbus, Ohio, United States of America; 3 Department of Bioengineering, University of California, San Diego, La Jolla, California, United States of America; 4 Department of Biomedical Engineering, Rutgers University, Piscataway, New Jersey, United States of America; National Research Council, ITALY

## Abstract

Pure tense (T) and relaxed (R) quaternary state polymerized human hemoglobins (PolyhHbs) were synthesized and their biophysical properties characterized, along with mixtures of T- and R-state PolyhHbs. It was observed that the oxygen affinity of PolyhHb mixtures varied linearly with T-state mole fraction. Computational analysis of PolyhHb facilitated oxygenation of a single fiber in a hepatic hollow fiber (HF) bioreactor was performed to evaluate the oxygenation potential of T- and R-state PolyhHb mixtures. PolyhHb mixtures with T-state mole fractions greater than 50% resulted in hypoxic and hyperoxic zones occupying less than 5% of the total extra capillary space (ECS). Under these conditions, the ratio of the pericentral volume to the perivenous volume in the ECS doubled as the T-state mole fraction increased from 50 to 100%. These results show the effect of varying the T/R-state PolyhHb mole fraction on oxygenation of tissue-engineered constructs and their potential to oxygenate tissues.

## Introduction

A major challenge in tissue engineering is provision of physiologically relevant oxygenation to cells cultured within tissue-engineered constructs [1]. Perfusion/static culture solutions without an O_2_ carrier cannot adequately oxygenate tissue-engineered constructs without the presence of significant hypoxic or hyperoxic regions [2]. A suitable alternative may consist of red blood cell (RBC) perfusion of the tissue culture in order to improve tissue oxygenation. Unfortunately, RBC perfusion may be plagued with issues ranging from short *ex vivo* storage shelf-life (i.e. 42 days) [3], limited supply [4,5], risk of transmission of unidentified pathogens [6], and RBC hemolysis [7]. In light of these challenges, hemoglobin (Hb)-based oxygen (O_2_) carriers (HBOCs) have emerged as promising candidates for use as universal RBC substitutes in tissue engineering applications [8–13].

Our group has synthesized variable molecular weight (MW) HBOCs with low and high O_2_ affinities [14–18] for use as RBC substitutes. These materials are based on glutaraldehyde polymerization of Hb in either the low O_2_ affinity (i.e. tense (T)) or high O_2_ affinity (i.e. relaxed (R)) quaternary state. In these studies, the T- or R-state PolyHbs either have low or high O_2_ affinity, however many applications exist where it may be desirable to tune the O_2_ affinity of the PolyHb solution to facilitate targeted O_2_ delivery based on varying oxygenation requirements of tissues.

The current study expands upon the work of Zhang et al. [17] and Zhou et al. [18], who synthesized and characterized the biophysical properties of bovine and human PolyHbs in either T- or R-state. In this study, pure T- and R-state polymerized human Hb (PolyhHb) solutions were synthesized, characterized and mixed at different molar ratios to yield PolyhHb mixtures with varying O_2_ affinities and biophysical properties. To assess the ability of the PolyhHb mixtures to oxygenate tissue engineered constructs, we developed a computational model of a single hollow fiber (HF) in a HF bioreactor housing hepatocytes (i.e. bio-artificial liver assist device), where the inlet partial pressure of O_2_ (pO_2_), mixture fraction, and total PolyhHb concentration were varied to assess oxygenation within the device. In vivo, the O_2_ tension gradient sensed by hepatocytes is thought to play an important role in the establishment of functional zonation along the acinus, which is integral to the proper functioning of the liver [19]. HF bioreactors mimic the microenvironment of a blood vessel via the continuously circulating media in the HF lumen that transports nutrients and O_2_ to the cells, while washing away metabolic waste products from the cells housed in the ECS. Therefore, this mathematical model can be used to assess the oxygenation potential of mixtures of T- and R-state PolyhHbs in tissue engineered constructs.

## Materials and methods

### Materials

Glutaraldehyde (50–70%), NaCl (sodium chloride), KCl (potassium chloride), NaOH (sodium hydroxide), Na_2_S_2_O_4_ (sodium dithionite), CaCl_2_.2H_2_O (calcium chloride), sodium lactate, N-acetyl-L-cysteine (NALC), NaCNBH_3_ (sodium cyanoborohydride), NaBH_4_ (sodium borohydride), Na_2_HPO_4_ (sodium phosphate dibasic), and NaH_2_PO_4_ (sodium phosphate monobasic) were procured from Sigma-Aldrich (St. Louis, MO). The HF tangential flow filtration (TFF) modules (rated pore sizes: 0.2 *μ*m, 50 nm, 500 kDa, and 100 kDa) were purchased from Spectrum Laboratories (Rancho Dominguez, CA). K_3_FeCN_6_ (potassium ferricyanide), KCN (potassium cyanide) and all other chemicals were purchased from Fisher Scientific (Pittsburgh, PA). Expired human RBC units were generously donated by Transfusion Services, Wexner Medical Center, The Ohio State University, Columbus, Ohio.

### Hb purification

Human Hb (hHb) was purified via TFF as described by Palmer et al. [20].

### Synthesis of PolyhHb

Deoxygenated and oxygenated hHb were polymerized with glutaraldehyde using methods developed previously [16,17] to yield 35:1 (molar ratio of glutaraldehyde to hHb) tense (T) and 30:1 relaxed (R) state PolyhHb, respectively.

To prepare 35:1 (molar ratio of glutaraldehyde to hHb) T-state (deoxygenated) PolyhHb, the hHb solution is devoid of dissolved O_2_ prior to and during the polymerization reaction. Presence of minute quantities of dissolved O_2_ will lead to formation of PolyhHb that is not exclusively in the T-state. To synthesize 30:1 R-state (oxygenated) PolyhHb, the Hb solution is fully saturated with O_2_ prior to and after the polymerization reaction to yield PolyhHb molecules exclusively in the R-state.

To generate completely deoxygenated hHb, 30–33 g freshly thawed hHb was diluted in PBS (0.1 M, pH 7.4) at room temperature in a total volume of 1200 mL. The diluted hHb solution was placed inside a sealed, air-tight glass bottle under continuous stirring. The glass bottle was placed in a water-bath to maintain the temperature of the hHb solution at 37°C. Long stainless steel needles were used to de-gas the hHb solution by alternate cycles of charging the headspace with N_2_ gas, and bubbling N_2_ through the hHb solution. After 35–45 min of degassing, samples were drawn from the bottle using a long stainless steel needle to measure the pO_2_ of the hHb solution using a Rapidlab 248 (Siemens, Malvern, PA) blood gas analyzer. When the measured pO_2_ of the hHb solution dropped to < 20 mm Hg, Na_2_S_2_O_4_ was added to the reaction vessel to remove residual O_2_ from the hHb solution [17]. 300 mg Na_2_S_2_O_4_ was dissolved in 300 mL N_2_ sparged PBS (0.1 M, pH 7.4) at room temperature, the Na_2_S_2_O_4_ solution was added to the hHb solution dropwise using a pump set to a flowrate of 0.1 mL/s. To confirm complete deoxygenation, the pO_2_ of the hHb solution was measured at the end of the titration process. Once the pO_2_ was out of range (pO_2_ < 0 mm Hg), an additional 200 mg Na_2_S_2_O_4_ was added to the hHb solution in 50 mg increments dissolved in 1 mL N_2_ sparged PBS in 5 minute intervals using a syringe.

Completely deoxygenated hHb was then polymerized using a 35:1 molar ratio of glutaraldehyde to hHb. The glutaraldehyde solution was prepared by diluting the necessary volume of glutaraldehyde with 5–10 mL of degassed PBS (0.1 M, pH 7.4). A 10 mL syringe was used to add the glutaraldehyde solution dropwise to the deoxygenated hHb under continuous stirring. The polymerization reaction was allowed to proceed at 37°C for 2 h in the absence of light under a N_2_ atmosphere.

**Fig 1:**
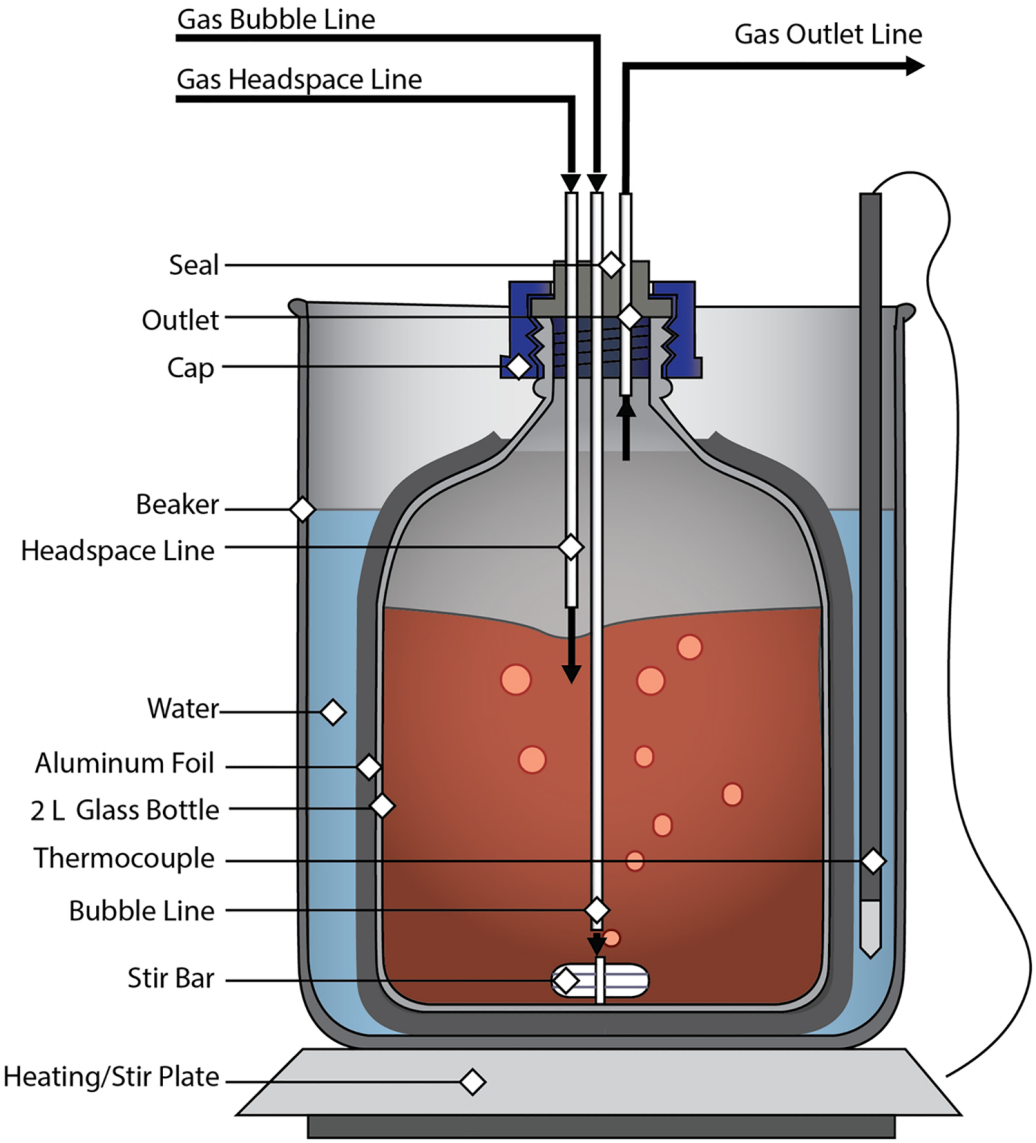
Schematic of the PolyhHb reactor vessel. This figure shows the glass bottle bench scale polymerization reactor used in our process to synthesize both the tense and relaxed state PolyhHb.

R-state hHb was prepared by saturating 1500 mL of a 20 mg/mL hHb solution with pure O_2_ gas for a period of 1–1.5 h at 37°C. Long stainless steel needles were used to oxygenate the hHb solution with alternate cycles of charging the headspace with O_2_ gas and bubbling O_2_ through the hHb solution. Complete O_2_-saturation of hHb solution was confirmed by measuring the pO_2_ of the hHb solution (pO_2_ > 749 mm Hg) [17]. Oxygenated hHb was then polymerized at a 30:1 molar ratio of glutaraldehyde to hHb. The glutaraldehyde solution was prepared by diluting the necessary volume of glutaraldehyde with 5–10 mL of oxygenated PBS (0.1 M, pH 7.4). A 10 mL syringe was used to add the glutaraldehyde solution dropwise to oxygenated hHb under continuous stirring. The polymerization reaction was allowed to proceed at 37°C for 2 h in the absence of light under an O_2_ atmosphere [17]. A schematic of the reactor setup used is shown in Fig 1.

To reduce the resultant Schiff bases and the methemoglobin (metHb) level of the PolyhHb solution, 6–8 mL of 6–8 M NaCNBH_3_ in PBS (0.1 M, pH 7.4) was added to the reaction vessel at the end of the reaction. The PolyhHb reaction vessel was placed in an ice-bath under continuous stirring for 30 min. Finally, 15–20 mL of freshly made 2 M NaBH_4_ was injected into the reaction vessel to reduce unreacted aldehydes. NaBH_4_ and NaCNBH_3_ were used in conjunction, since they reduce Schiff bases and free aldehyde in solution [16,17,21]. The pO_2_ of the hHb solution was monitored before and after polymerization to ensure that the polymerization reaction was carried out with hHb in the desired quaternary state (T- or R-state).

### Diafiltration of PolyhHb

**Fig 2:**
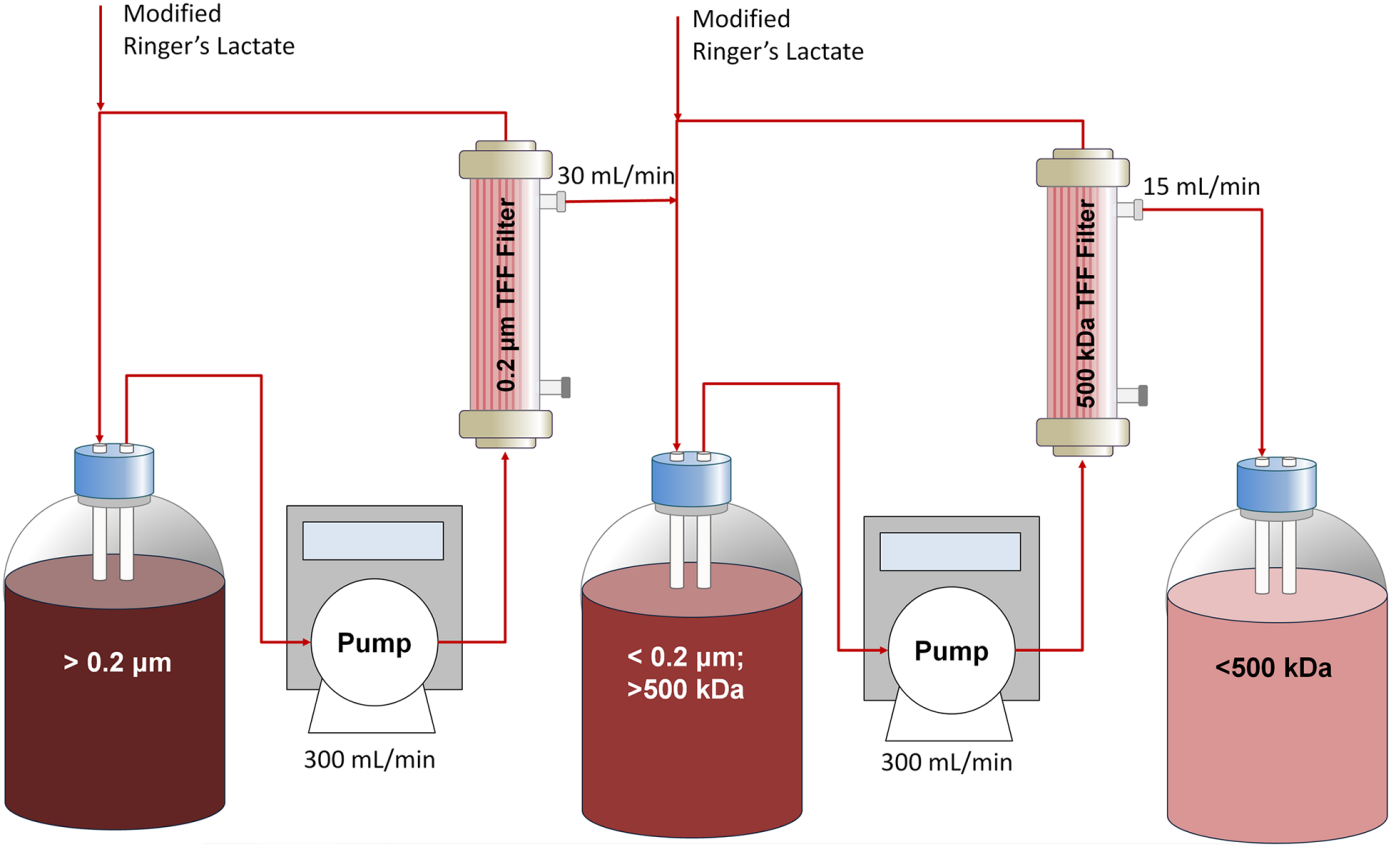
Diafiltration, purification, and concentration schematic. Shown in this figure is the multistage tangential flow filtration process for processing the polymerized PolyhHb.

Small hHb polymers, reduced glutaraldehyde, and excess quenching reagents were removed from the synthesized PolyhHb solutions using a diafiltration protocol developed in our lab. PolyhHb solutions were passed through 0.2 *μ*m TFF modules to remove large particles. PolyhHb solutions were then buffer exchanged in an isotonic modified Ringer’s lactate buffer (NaCl 115 mmol/L, KCl 4 mmol/L, CaCl_2_.2H_2_O 1.4 mmol/L, NaOH 13 mmol/L, sodium lactate 27 mmol/L, and NALC 12.3 mmol/L). The PolyhHb solutions were subjected to 8–9 cycles of diafiltration (4°C) on 500 kDa TFF modules at a 1:9 (v/v) ratio of PolyhHb to modified Ringer’s lactate buffer. Fig 2 shows a schematic of the diafiltration setup used in the study. This process was performed at 4°C under ambient air conditions.

The filtrate from the 9^th^ diafiltration cycle was collected and measured via UV-visible spectroscopy to verify complete removal of small hHb species. Buffer-exchanged PolyhHb solutions were concentrated on 500 kDa TFF cartridges (Spectrum Labs, Rancho Dominguez, CA). The resulting concentrated PolyhHb solutions were stored at -80°C. Sterile lab supplies were used for all experiments. All tubing, glassware, and filters were de-contaminated by immersing them overnight in 1 M NaOH and then rinsing thoroughly with double distilled de-ionized water.

### Preparation of T- and R-State PolyhHb mixtures

Stock solutions of 35:1 T-state PolyhHb and 30:1 R-state PolyhHb having the same molar concentration (on a heme basis) were prepared. These stock solutions were mixed at different molar ratios i.e. 0.25:0.75, 0.5:0.5, and 0.75:0.25 to yield mixtures of T- and R-state PolyhHbs.

### Hydrodynamic diameter of PolyhHb

The hydrodynamic diameter of PolyhHb was measured using a Zetasizer Nano Dynamic Light Scattering (DLS) spectrometer (Malvern Instruments Ltd., Worcestershire, United Kingdom) at 37°C [21]. The PolyhHb solutions were diluted to a final concentration of ~ 2 mg/mL using PBS (0.1 M, pH 7.4). An internal heating system and temperature controller maintained the sample temperature at 37°C [21].

### Methemoglobin level and protein concentration of hHb/PolyhHb

The cyanomethemoglobin method was used to measure the methemoglobin (metHb) level of hHb/PolyhHb solutions [22,23]. The Bradford assay was performed using the Coomassie Plus protein assay kit (Pierce Biotechnology, Rockford, IL) to estimate the total protein concentration in solution [17,24].

### O_2_–hHb/PolyhHb equilibria measurements

O_2_-hHb/PolyhHb equilibrium binding curves were generated using a Hemox Analyzer (TCS Scientific Corp., New Hope, PA) at 37°C (physiological temperature) as described in the literature. The Hill equation (Eq 1) was used to fit the OEC obtained for hHb/PolyhHb [22,25].

$$\text{Y}=\frac{\text{Abs}-{\text{A}}_{0}}{{\text{A}}_{\infty }-{\text{A}}_{\text{0}}}=\frac{{\text{pO}}_{2}{}^{\text{n}}}{{\text{pO}}_{2}{}^{\text{n}}-{\text{p}}_{50}{}^{\text{n}}}$$ (1)

Where *Abs* is the measured absorbance of the sample, *A*_0_ and *A*_∞_ correspond to the sample absorbance at 0 mm Hg and at maximum saturation, respectively. The *P*_*50*_ (partial pressure of O_2_ at which 50% of the hHb/PolyhHb is saturated with O_2_) and the cooperative coefficient (*n*) of hHb/PolyhHb were regressed by fitting the OECs to Eq 1 [25].

### Rapid kinetic measurements of hHb/PolyhHb solutions

hHb/PolyhHb gaseous ligand binding/release kinetics were measured using an Applied Photophysics SF-17 microvolume stopped-flow spectrophotometer (Applied Photophysics Ltd., Surrey, United Kingdom). Rapid kinetic measurements were performed using protocols previously described by Rameez and Palmer [10,11,26]. For all stopped-flow measurements, a control of hHb was used to ensure the authenticity of the results. PBS (0.1 M, pH 7.4) was used as the reaction buffer for all kinetic measurements. Flash photolysis mediated O_2_ association kinetics were measured using procedures described by Olsen et al. [27]. Prior to flash photolysis, each sample was oxygenated by placing it under 1 atm of O_2_ for 15 minutes. Complete oxygenation was verified by spectral analysis. Flash photolysis was performed on 12.75 μM (on a per heme basis) samples with an excitation wavelength of 425 nm (3.5 mJ/pulse). Samples were excited with a Q-switch Nd-YAG laser (Spectra Physics, Santa Clara, CA) pumped optical parametric oscillator (Spectra Physics, Santa Clara, CA) that delivered pulses of *ca*. 8 ns at 10 Hz. The pulse energy was about 3.5 mJ/pulse at the sample. The spectrometer (Edinburgh Instruments LP980, Livingston, UK) used a 150 W Xe-lamp to generate probe light at a 90 degree angle from the pump laser. Kinetic traces were recorded by PMT and a digital oscilloscope, while transient spectra were collected with a CCD (Andor Technology, Belfast, UK). O_2_ association was monitored at 430 nm. Complete photolysis of O_2_ was verified for each sample.

### Computational methods

**Fig 3:**
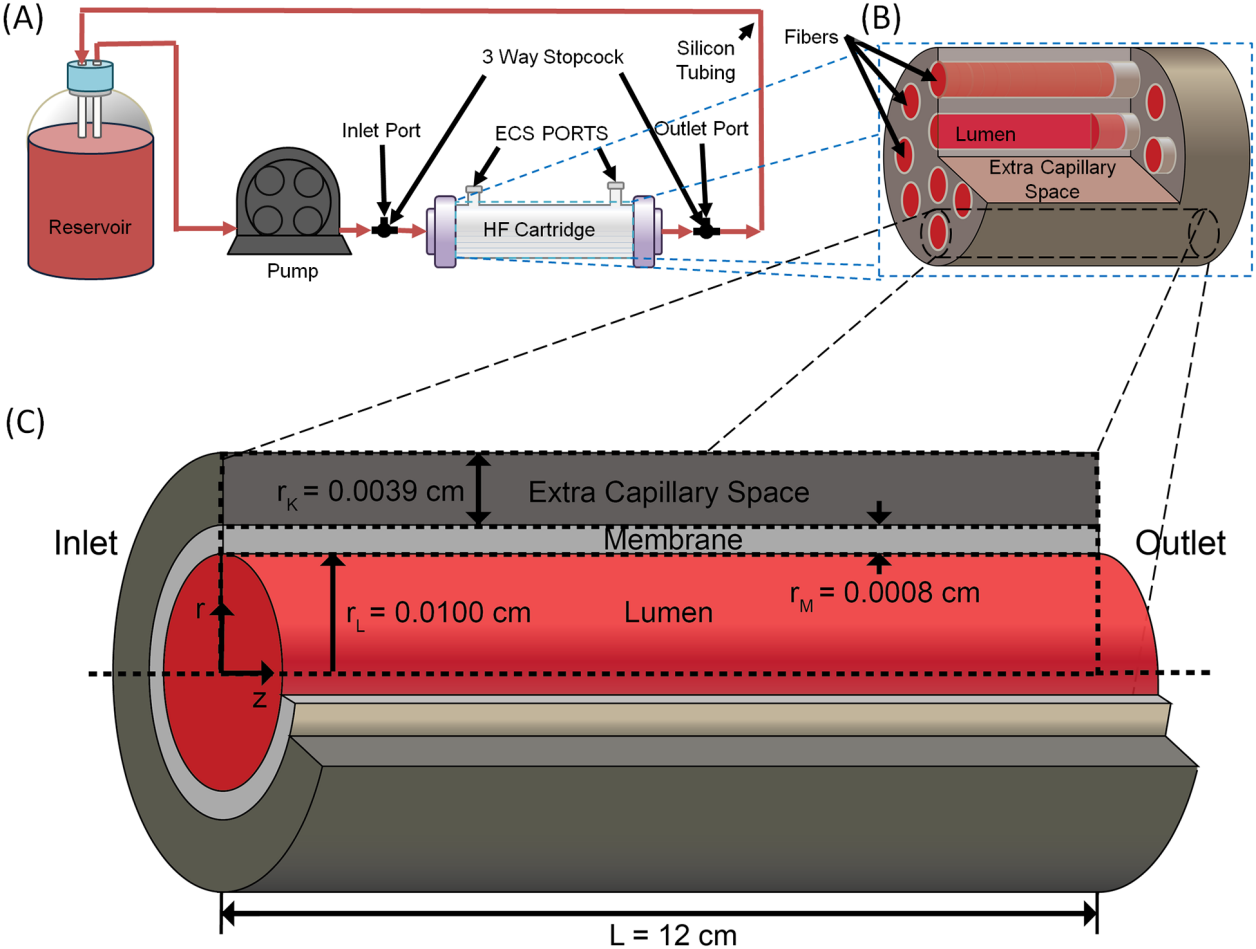
Schematic of the HF bioreactor system and individual HF model geometry. Shown here is the basic schematic of hemoglobin solutions pumped through a HF reactor with uniformly distributed fibers with the shown geometry.

To assess the ability of mixtures and T- and R-state PolyhHb to oxygenate tissue engineered constructs, we computationally evaluated O_2_ transport in a single fiber of a HF bioreactor housing hepatocytes (i.e. bio-artificial liver assist device). This type of device can be used to replicate various liver functions, and has been used as an artificial liver assist device to support patients with failing livers [28]. The HF bioreactor modeled in this study consists of a Spectrum Laboratories (Rancho Dominguez, CA) commercial HF bioreactor (cat.#400–011) containing 2,205 individual polyethylene fibers. The HF membrane has a 35 kDa MW cut-off which prevents PolyhHb (M_w_ > 35 kDa) transport out of the lumen into the extra capillary space (ECS). The ECS houses cultured hepatocytes. The O_2_ concentration profile was modeled with a modified form of the Krogh tissue cylinder model. This model consists of three subdomains: a cylinder representing the lumen, an annulus representing the membrane, and an outer annulus representing the ECS. A mixture of cell culture media and PolyhHb flows through the lumen to provide nutrients and remove waste to/from the cells which reside entirely within the ECS. A schematic of the HF bioreactor system and individual HF model geometry is shown in Fig 3.

Additional information about this model and the physical constants used can be found in the literature [29]. The model is evaluated with finite element analysis in Comsol Multiphysics (Version 4.3, Comsol, Inc., Burlington, MA). Pressure and velocity profiles were first evaluated independently. Mass conservation equations for O_2_, and the HBOCs were then solved simultaneously. Inlet pO_2_, total HBOC concentration, and HBOC fraction were varied during simulation by 0–140 mm Hg, 0–130 mg/mL and 1–100% R-state respectively.

## Results and discussion

### PolyhHb synthesis and characterization

It is necessary to measure the biophysical properties of 35:1 T-state PolyhHb, 30:1 R-state PolyhHb, and various mixtures of these two types of HBOCs to evaluate their O_2_ transport potential in transfusion and tissue engineering applications. Table 1 compares the biophysical properties (hydrodynamic diameter, protein concentration, % metHb, O_2_ equilibria, and gaseous ligand binding/release kinetics) of hHb, 35:1 T-state PolyhHb and 30:1 R-state PolyhHb. Table 1 also compares the biophysical properties of molar mixtures of 35:1 T-state PolyhHb and 30:1 R-state PolyhHb. In this comparison, one batch of 35:1 T-state PolyhHb and one batch of 30:1 R-state PolyhHb were selected to formulate the mixtures. Therefore, entries in Table 1 do not have error bars.

**Table 1:** Biophysical properties of PolyhHb.

Property	hHb (n = 15)	35:1 T-state PolyhHb (n = 15)	30:1 R-state PolyhHb (n = 15)	35:1 T-state PolyhHb	T-state Mole Fraction			30:1 R-state PolyhHb
					.75	0.5	0.25	
**Diameter (nm)**	5.5 ^39^^a^^b^	93.78 ± 16.34^c^	87.17 ± 12.72^c^	102.7	98.1	96.2	92.4	90.1
**[Hb] (g/dL)**	31.47 ± 7.92^a^^b^	11.04 ± 1.13^c^	11.72 ± 1.44^c^	10.79	10.79	10.79	10.79	10.79
**MetHb (%)**	0^a^^b^	5.08 ± 0.55^b^^c^	3.49 ± 1.23^a^^c^	4.97	4.78	4.53	4.66	5.40
**P_50_ (mm Hg)**	12.40 ± 1.04^a^^b^	37.35 ± 7.91^b^^c^	1.96 ± 0.77^a^^c^	32.35	23.54	15.07	4.24	1.77
**Cooperativity (*n*)**	2.62 ± 0.10^a^^b^	0.79 ± 0.11^b^^c^	1.10 ± 0.17^a^^c^	0.70	0.75	0.53	0.53	0.97
**k_off, O2_ (s^-1^)**	37.06 ± 2.92^a^^b^	47.35 ± 4.15^b^^c^	23.98 ± 4.11^a^^c^	41.47	29.88	25.69	18.35	19.42
**k_on, O2_ (μM^-1^s^-1^)**	40.85 ± 1.49 ^a^^b^	28.59 ± 0.66^b^^c^	12.12 ± 0.69^a^^c^	-	-	-	-	-
**k_on, CO_ (μM^-1^s^-1^)**	0.199 ± 0.01^a^	0.12 ± 0.02^b^^c^	0.20 ± 0.05^a^	0.10	0.14	0.15	0.15	0.24
**k_ox, NO_ (μM^-1^s^-1^)**	35.99 ± 6.06^a^^b^	13.21 ± 4.66^c^	14.57 ± 3.57^c^	13.22	12.59	12.25	13.04	10.26

The error bars represent the standard deviation from 15 replicates. One batch of 35:1 T-state PolyhHb and one batch of 30:1 R-state PolyhHb were used to formulate mixtures. Therefore, the entries in this table do not have error bars.

^a^ p < 0.05 compared with 35:1 T-state PolyhHb

^b^ p < 0.05 compared with 30:1 R-state PolyhHb

^c^ p < 0.05 compared with hHb.

### Hydrodynamic diameter of PolyhHb

The hydrodynamic diameter of 35:1 T-state PolyhHb (93.78 ± 16.34 nm) was not significantly different (p<0.05) compared to the diameter of 30:1 R-state PolyhHb (87.17 ± 12.72 nm). However, the measured particle diameter for mixtures of T- and R-state PolyhHbs was proportional to the molar ratio of pure T-state and pure R-state PolyhHb. In contrast, the diameter of T- and R-state PolyhHbs was significantly (p<0.05) larger than the diameter reported in the literature for cell-free hHb (~5.5 nm) [30]. Therefore, the large molecular radius of T- and R-state PolyhHbs can avoid the side-effects associated with transfusion of cell-free Hb such as unfolding of the globin chain leading to the release of cytotoxic free-heme and renal toxicity, dissociation of tetrameric Hb into αβ dimers and extravasation through the blood vessel wall into the surrounding tissue space leading to oxidative tissue injury, and scavenging of endothelial NO leading to vasoconstriction and systemic hypertension [10,11,31–33].

### MetHb level and protein concentration of PolyhHb

The metHb level of 35:1 T-state PolyhHb was significantly (p<0.05) higher than 30:1 R-state PolyhHb (Table 1). However, metHb levels for the mixtures showed no trend with mixture ratio. R-state PolyhHb was synthesized in an oxygenated environment, and intuitively expected to yield higher metHb levels compared to T-state PolyhHb. However, the opposite was observed. This can be explained by the duration of hHb deoxygenation before polymerization. For R-state PolyhHb, the polymerization reaction is initiated after 1–1.5 h of oxygenation while in T-state it is 2 hours after deoxygentation. The extended period of time hHb is maintained at 37°C during the deoxygenation step leads to higher metHb levels for T-state PolyhHb.

Protein concentrations for 35:1 T-state and R-state PolyhHb solutions ranged between 11.04 ± 1.13 and 11.72 ± 1.44 g/dL, respectively. These concentrations are comparable to the Hb concentration in whole blood (15.7 g/dL for men and 13.8 g/dL for women) [34]. Additionally, these concentrations are comparable to the Hb concentrations reported in the literature for commercial HBOCs: HBOC-201^®^ ([Hb] ~ 13 g/dL), PolyHeme^®^ ([Hb] ~ 10 g/dL) [35], and Hemolink^®^ ([Hb] ~ 9.7 g/dL) [36].

### O_2_–hHb/PolyhHb equilibria

**Fig 4:**
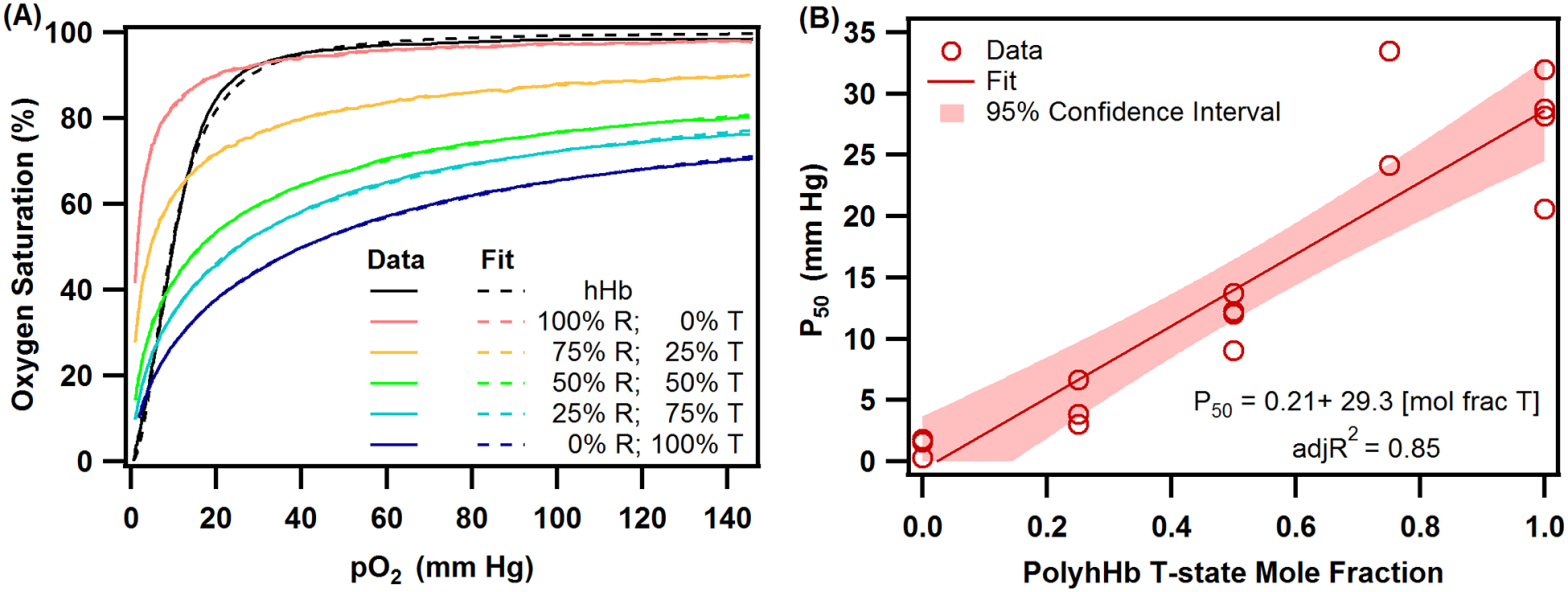
hHb and PolyhHb O_2_ equilibrium. (A) Comparison of O_2_ equilibrium curves of hHb, 30:1 R-state PolyhHb, 35:1 T-state PolyhHb, and mixtures of 35:1 T-state PolyhHb and 30:1 R-state PolyhHb at various molar fractions of T: 0.75,0.5 and 0.25. Dots represent experimental data and the corresponding solid lines of the same color represent curve fits. (B) Dependence of P_50_ on mole fraction of 35:1 T-state PolyhHb to 30:1 R-state PolyhHb. The data were fit to a linear function using JMP 9.2.

The O_2_-hHb/PolyhHb equilibrium data were fit to the Hill equation (Eq 1) to regress the *P*_*50*_ and cooperativity coefficient (*n*). Unlike hHb, the shape of the equilibrium O_2_ binding curves obtained for PolyhHbs are not sigmoidal. This indicates a significant loss in cooperative binding of O_2_ to Hb in PolyhHbs compared to unmodified hHb. These observations are shown in Fig 4(A), which compares typical O_2_ equilibrium curves of hHb, 30:1 R-state PolyhHb, 35:1 T-state PolyhHb, and mixtures of 35:1 T-state PolyhHb and 30:1 R-state PolyhHb at molar ratios of 0.75:0.25, 0.50:0.50 and 0.25:0.75. Dots represent experimental data and corresponding solid lines of the same color represent curve fits.

The 35:1 T-state PolyhHb exhibited lower O_2_ affinity (*P*_*50*_ ~ 37.35 ± 7.91 mm Hg) compared to hHb (*P*_*50*_ ~ 12.40 ± 1.04 mm Hg). Polymerization of Hb in the deoxy-state limits the resultant PolyhHb to the T-state quaternary conformation compared to native Hb, thereby accounting for its higher *P*_*50*_ [17,37]. In contrast, high O_2_ affinity (*P*_*50*_ ~ 1.96 ± 0.77 mm Hg) was observed for 30:1 R-state PolyhHb compared to hHb and T-state PolyhHb. Polymerization of Hb in the oxygenated-state limits the resultant PolyhHb to the R-state quaternary conformation thereby accounting for its’ lower *P*_*50s*_ [17]. The literature suggests that HBOCs with high *P*_*50s*_ target O_2_ transport to the systemic circulation, while HBOCs with low *P*_*50s*_ target O_2_ transport to the peripheral tissues via microcirculation [37]. Furthermore, mixtures of HBOCs with varying O_2_ affinities might be a suitable option for restoring tissue oxygenation during resuscitation from hemorrhagic shock [15]. Therefore in this study, we evaluated *in silico* the ability of mixtures of 35:1 T-state PolyhHb and 30:1 R-state PolyhHb at molar ratios of 0.75:0.25, 0.50:0.50, and 0.25:0.75 to supply and regulate O_2_ levels in a single fiber of a HF hepatic bioreactor. We observed that the *P*_*50*_ for various mixtures of T- and R-state PolyhHbs were proportional to the molar ratio of pure T-state and pure R-state PolyhHb (Table 1). Fig 4(B) shows the dependence of *P*_*50*_ on molar ratio of 35:1 T-state to 30:1 R-state PolyhHbs. These data were fit to a linear function using JMP 9.2.

Both 35:1 T-state and 30:1 R-state PolyhHbs display lower cooperativity (*n*) compared to unmodified hHb (*n* ~ 2.62 ± 0.10) **(**Table 1). The quaternary conformational changes observed in a Hb molecule during its transition from the deoxy- to the oxy-state involve rotation of the two symmetrical αβ dimers by 15° relative to each other and a translation of 0.1 nm along the rotation axis [38]. This rotation about the axis is perhaps hindered by the inter- and intra-molecular glutaraldehyde cross-links in PolyhHb molecules, thus resulting in the observed low cooperativities [21].

### Rapid kinetic measurements of hHb/PolyhHb solutions

The kinetics of PolyhHbs with physiological relevant gaseous ligands were measured to compare their ligand binding/release kinetics. These rates are important to evaluate the ability of these particles to store and transport important gaseous ligands such as O_2_, CO, and NO. NO dioxygenation kinetics can predict the ability of PolyhHb to scavenge NO, which is the major mechanism for the development of vasoconstriction and systemic hypertension. O_2_ dissociation measurements can be linked to autoregulation theory for the development of vasoconstriction and systemic hypertension [10].

### Reactions with O_2_

**Fig 5:**
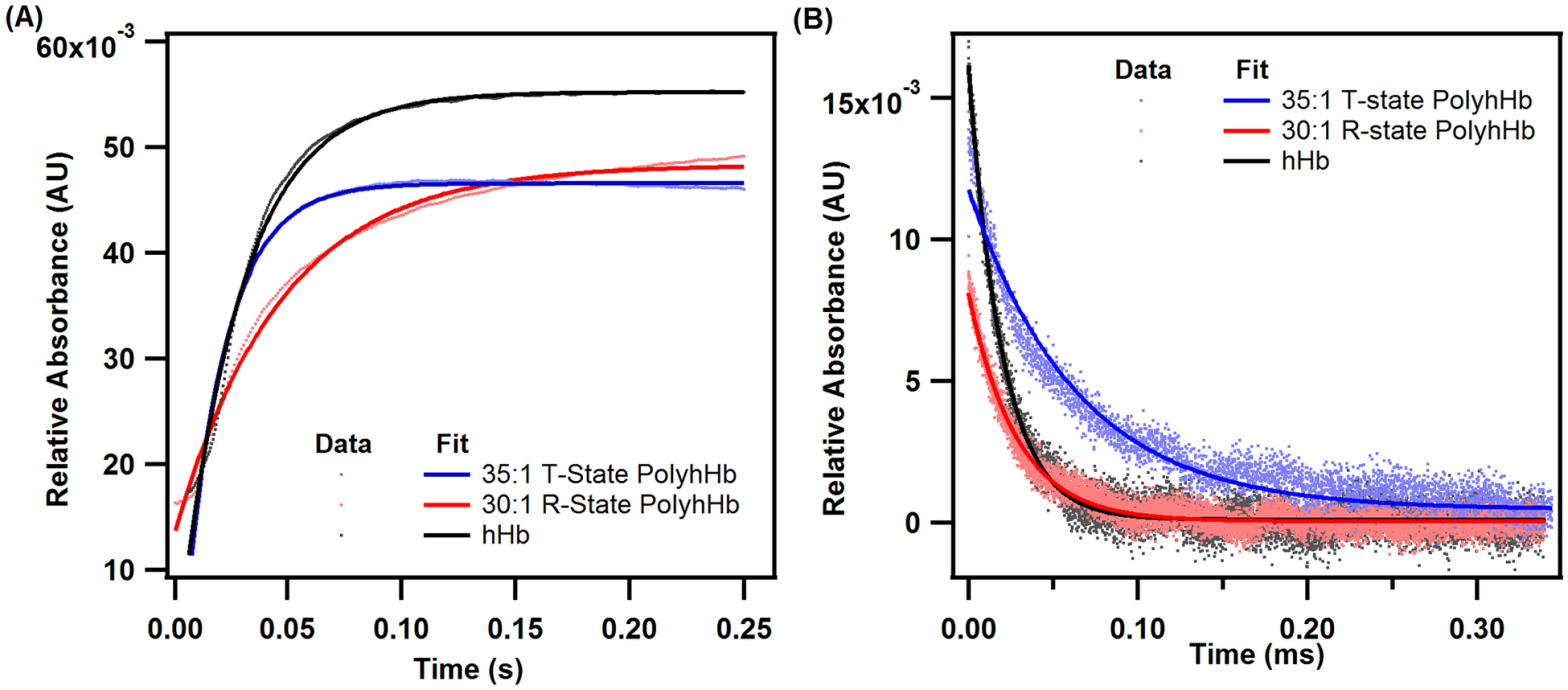
Deoxygenation and oxygenation kinetic time courses for hHb, T-state PolyhHb and R-state PolyhHb. (A) Comparison of time courses for deoxygenation in the presence of 1.5 mg/mL sodium and (B) oxygenation time course facilitated by flash photolysis of hHb (red), 30:1 R-state PolyhHb (black), and 35:1 T-state PolyhHb (blue). Dots represent experimental data and the corresponding solid lines of the same color represent curve fits. The experimental data shows an average of 10–15 kinetic traces. For deoxygenation, the reactions were monitored at 437.5 nm and 20°C. For oxygenation, the reactions were monitored at 430 nm. PBS (0.1 M, pH 7.4) was used as the reaction buffer.

${\text{k}}_{{\text{off,O}}_{\text{2}}}$ for 35:1 T-state PolyhHb (47.35 ± 4.15 s^-1^) was significantly (p<0.05) higher than that obtained for 30:1 R-state PolyhHb (23.98 ± 4.11 s^-1^) and unmodified hHb (37.06 ± 2.92 s^-1^). Also, ${\text{k}}_{{\text{off,O}}_{\text{2}}}$ values obtained for R-state PolyhHb are significantly lower (p<0.05) than the rate constant obtained for unmodified hHb. Furthermore, we observed that ${\text{k}}_{{\text{off,O}}_{\text{2}}}$ for various mixtures of T- and R-state PolyhHbs increased with increasing molar ratio of 35:1 T-state to 30:1 R-state PolyhHb (Table 1). These observations are consistent with the literature and are expected given the contrasting O_2_ affinities of T- and R-state PolyhHbs [15,17]. ${\text{k}}_{{\text{on,O}}_{\text{2}}}$ for 35:1 T-state PolyhHb (12.12 ± 0.69 s^-1^μM^-1^) was significantly (p<0.05) lower than that obtained for 30:1 R-state PolyhHb (28.59 ± 0.66 s^-1^μM^-1^) and unmodified hHb (40.85 ± 1.49 s^-1^μM^-1^). In addition, ${\text{k}}_{{\text{on,O}}_{\text{2}}}$ values obtained for 30:1 R-state PolyhHb are significantly (p<0.05) lower than that obtained for unmodified hHb. In these flash photolysis experiments, we observed that the rate of O_2_ binding to T-state PolyhHb was significantly lower than unmodified hHb. This can be explained by incomplete O_2_ binding to T-state PolyhHb even under 1 atm of pure O_2_. At equilibrium and at this dissolved O_2_ concentration (pO_2_ ~740 mm Hg), 35:1 T-state PolyhHb is only ~95% saturated with O_2_. However, ${\text{k}}_{{\text{on,O}}_{\text{2}}}$ for unmodified hHb was similar to reported literature values [39,40]. Interestingly, ${\text{k}}_{{\text{on,O}}_{\text{2}}}$ for 35:1 T-state PolyhHb was on the same order of magnitude compared to chemically modified T-state hHb (5–10 s^-1^μM^-1^) [27]. However, ${\text{k}}_{{\text{off,O}}_{\text{2}}}$ for 35:1 T-state PolyhHb was drastically different compared to chemically modified T-state hHb ${\text{k}}_{{\text{off,O}}_{\text{2}}}$(~500–1000 s^-1^) [27]. For 30:1 R-state PolyhHb, ${\text{k}}_{{\text{off,O}}_{\text{2}}}$ was similar to the values reported for R-state hHb (~20 s^-1^) [27]. In contrast, the value for ${\text{k}}_{{\text{on,O}}_{\text{2}}}$ for 35:1 R-state PolyhHb was dramatically different compared to R-state hHb (~66 s^-1^μM^-1^) [27]. Reduced interactions between neighboring globin subunits in T- and R-state PolyhHb compared to non-polymeric R- and T-state hHb may result in the observed deviations for PolyhHb O_2_ association and dissociation kinetics when the PolyhHb is not in the thermodynamically preferred conformational state (i.e. fully deoxygenated T-state PolyhHb or fully oxygenated R-state PolyhHb). Unfortunately, the random and extensive nature of the glutaraldehyde crosslinks precludes any higher-level analysis of this behavior. Similar effects are further demonstrated in the reduced NO dioxygenation reaction rate constant for the PolyhHbs compared to hHb. Fig 5 compares typical kinetic time courses of O_2_ dissociation and association for hHb, 35:1 T-state PolyhHb and 30:1 R-state PolyhHb.

The high O_2_ offloading rate of cell-free hHb forms the basis of autoregulation theory that explains the development of vasoconstriction and systemic hypertension upon transfusion of HBOCs [41–43]. Thus, moderate O_2_ release rates are critical in improving HBOC efficacy. Therefore, the PolyhHbs and their mixtures synthesized in our lab can potentially deliver O_2_ to ischemic tissues at regulated rates potentially avoiding vasoconstriction resulting from the oversupply of O_2_.

### Reactions with CO

**Fig 6:**
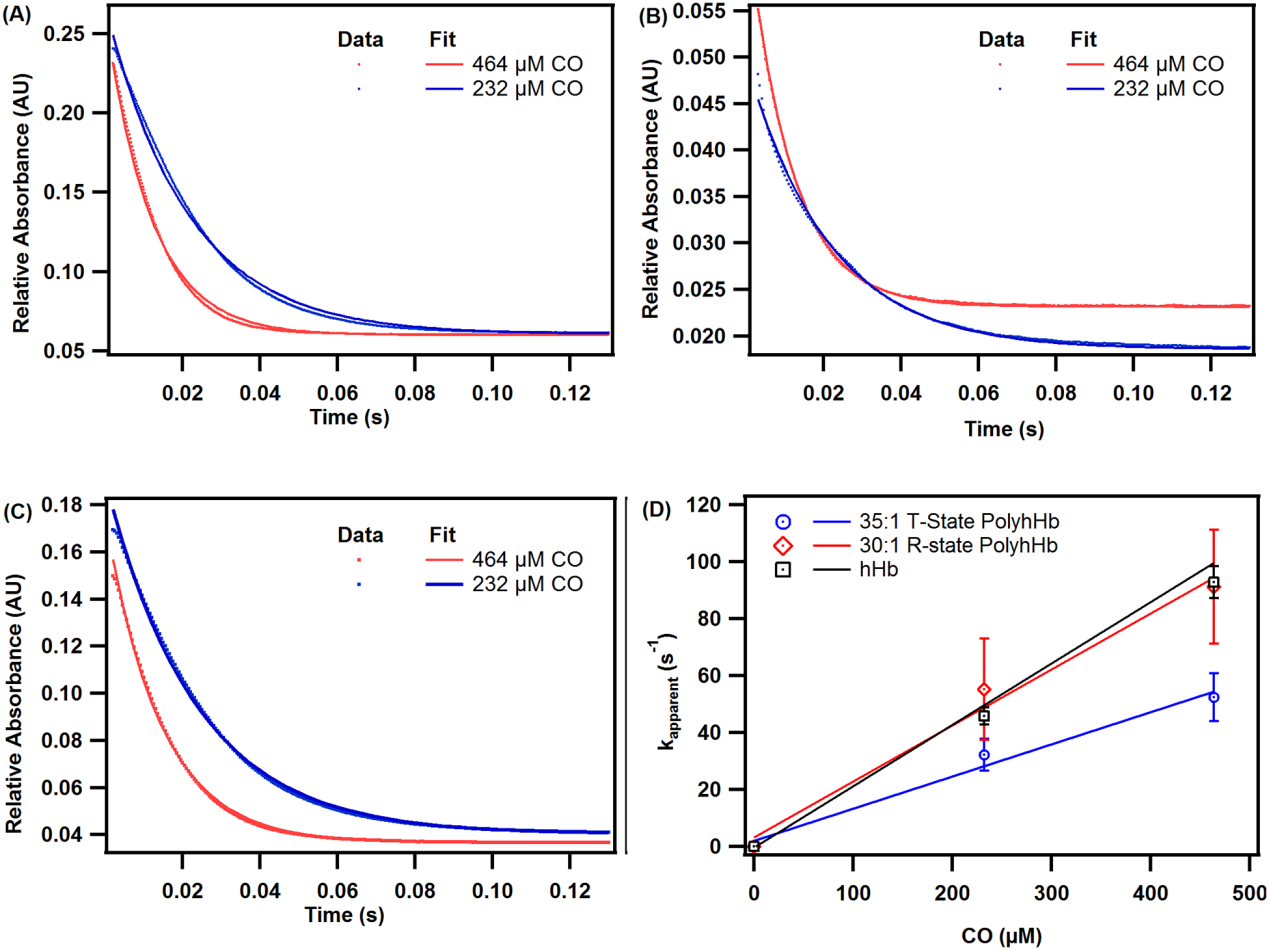
Time courses for the CO association reaction. Time courses for the CO association reaction with deoxygenated (A) hHb, (B) 30:1 R-state PolyhHb, and (C) 35:1 T-state PolyhHb. Dots represent experimental data and the corresponding solid lines of the same color represent curve fits. Experimental data shows an average of 10–15 kinetic traces. The reactions were monitored at 437.5 nm and 20°C. PBS (0.1 M, pH 7.4) was used as the reaction buffer. (D) Comparison of CO association rates of hHb, 35:1 T-state PolyhHb, and 30:1 R-state PolyhHb. The error bars represent the standard deviation from 15 replicates.

Fig 6 shows characteristic CO association kinetic time courses for deoxygenated hHb (**A**), 30:1 R-state PolyhHb (**B**), and 35:1 T-state PolyhHb (**C**). The dependence of the pseudo first-order rates on CO concentration for hHb, 35:1 T-state PolyhHb, and 30:1 R-state PolyhHb is shown in panel **D**. Therefore, the slopes of the linear fits in panel **D** indicate the second-order CO association rate constants reported in Table 1.

The k_on_,_CO_ rates obtained for unmodified hHb and 30:1 R-state PolyhHb evaluated in this study are significantly higher (p<0.05) than the values obtained for 35:1 T-state PolyhHb (Table 1). Similar findings have been reported in the literature and suggest that polymerization of Hb in the T-state limits heme pocket accessibility to CO [15]. Moreover, polymerization of Hb in the R-state results in more open conformation and greater heme pocket accessibility. This explains the higher k_on_,_CO_ rate constant observed for 30:1 R-state PolyhHb [15]. The k_on_,_CO_ rate constants for the T- and R-state molar mixtures showed no trend with mixture ratio (Table 1).

### Reactions with NO

**Fig 7:**
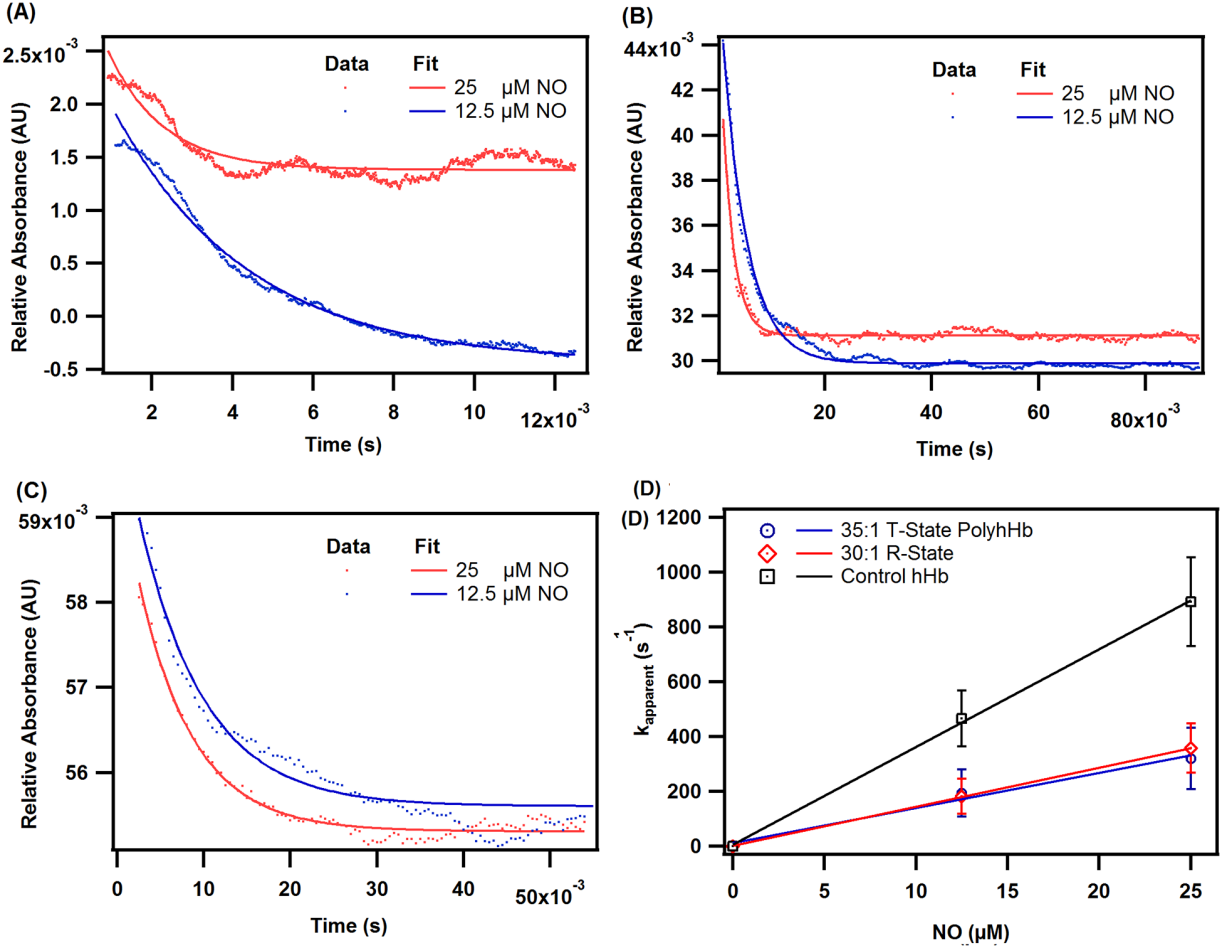
Time courses for the NO deoxygenation reaction. Time courses for the NO dioxygenation reaction with oxygenated (A) hHb, (B) 30:1 R-state PolyhHb, and (C) 35:1 T-state PolyhHb. Dots represent experimental data and the corresponding solid lines of the same color represent curve fits. Experimental data shows an average of 7–10 kinetic traces. The reactions were monitored at 420 nm and 20°C. PBS (0.1 M, pH 7.4) was used as the reaction buffer. (D) Comparison of NO dioxygenation rates of hHb, 35:1 T-state PolyhHb, and 30:1 R-state PolyhHb. The error bars represent the standard deviation from 15 replicates.

Fig 7 shows characteristic NO dioxygenation kinetic time courses for oxygenated hHb (**A**), 30:1 R-state PolyhHb (**B**), and 35:1 T-state PolyhHb (**C**). The dependence of the pseudo first-order rates on NO concentration for hHb, 35:1 T-state PolyhHb, and 30:1 R-state PolyhHb is shown in panel **D**. Therefore, the slopes of the linear fits in panel **D** are used to calculate the second-order NO dioxygenation rate constants reported in Table 1. The NO dioxygenation rate constant for 35:1 T-state PolyhHb was comparable (p>0.05) to the rate constant for 30:1 R-state PolyhHbs (Table 1). Similar k_oX_,_NO_ values have been reported in the literature [15,17]. The k_oX_,_NO_ for molar mixtures of T- and R-state PolyhHb showed no trend with mixture ratio.

### Comparison to commercial HBOCS

Comparisons (wherever possible) were made between the biophysical properties of T- and R-state PolyhHbs synthesized in this study and commercial HBOCs. The biophysical properties of selected commercial HBOCs are shown in Table 2. The T- and R-state PolyhHbs synthesized in this study have significantly larger diameters compared to the computed diameters [44] of Oxyglobin^®^ (Biopure Corp, Cambridge, MA, USA) and Hemolink^®^ (Hemosol Inc., Toronto, Canada) [45,46]. MetHb values observed for the T- and R-state PolyhHbs synthesized in our lab are comparable to those reported for HBOC-201^®^, PolyHeme^®^ (Northfield Laboratories Inc., Northfield, IL, USA) [35], and Hemolink^®^ [36]. The *P*_*50s*_ of 35:1 T-state PolyhHbs are in agreement with the *P*_*50*_ values reported in the literature for commercial HBOCs, HBOC-201^®^, PolyHeme^®^ [35], and Hemolink^®^.The cooperativity values of the T- and R-state PolyhHbs are comparable to the reported values HBOC-201^®^ [35] and Hemolink^®^ [36]. The observed *n* values are slightly lower than those reported for PolyHeme^®^ [35]. The ${\text{k}}_{{\text{off,O}}_{\text{2}}}$ values for T- and R-state PolyhHbs are lower than the deoxygenation rate constants reported in the literature for Hemolink^®^ [47], and Oxyglobin^®^ [46]. The k_on_,_CO_ values for T-state PolyhHbs are comparable to those reported in the literature for Hemolink^®^ [47], but are significantly lower than the values recorded for Oxyglobin^®^ [46]. In contrast, CO association rate constants for R-state PolyhHbs are comparable to Oxyglobin^®^, but are significantly higher than Hemolink^®^.

**Table 2:** Biophysical properties of commercially available HBOCs.

HBOC	Effective Diameter (nm)	P_50_ (mm Hg)	n	Met (%)	${\text{k}}_{{\text{off,O}}_{\text{2}}}$ s^-1^	k_on_,_CO_ μM^-1^s^-1^
*Oxyglobin^®^*	5.85–10.49 [44]	38.4 [48]	1.4 [48]	3.68–5.68 [49]	61.8 ± 1.6 [46]	0.19 ± 0.02 [46]
*Hemolink^®^*	5.28–11.13 [44]	~33.5 [36]	~0.95 [36]	~ 6.6 [36]	130 ± 3.5 [47]	0.12 ± 0.04 [47]
*HBOC-201^®^*	5.73–11.10 [50]	~38 [35]	~1.4 [35]	< 10 [35]	NA	NA
*PolyHeme^®^*	5.59–10.31 [50]	~29 [35]	~1.7 [35]	< 8 [35]	NA	NA

### Computational results

The measured biophysical properties of pure T- and R-state PolyhHb solutions (Table 1) were incorporated into a computational model describing O_2_ transport in a single fiber of a hepatic HF bioreactor where the inlet pO_2_, mixture fraction, and total PolyhHb concentration were varied.

**Fig 8:**
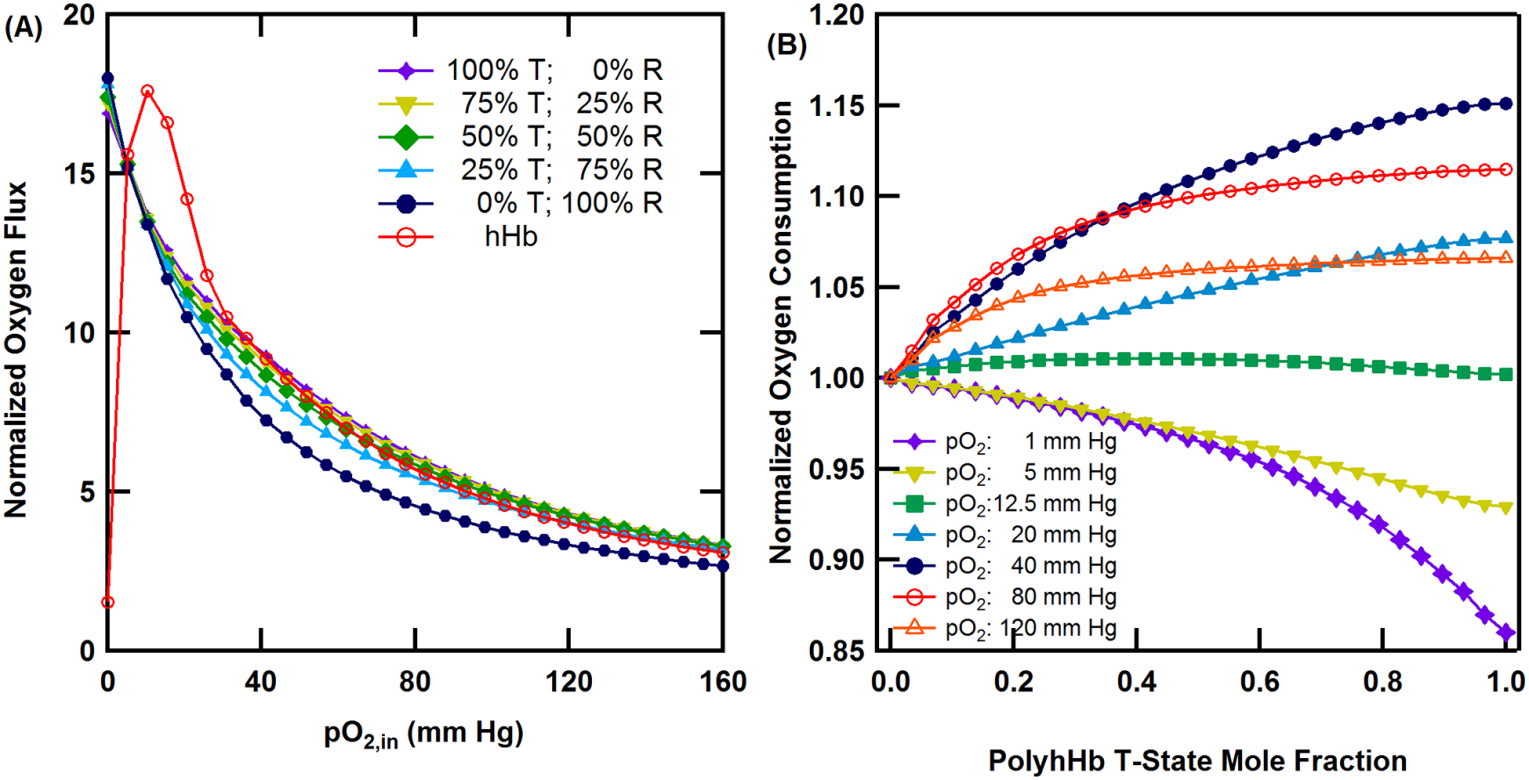
Effects of PolyhHb mixtures and hHb on oxygenation of the HF bioreactor. (A) Unsupplemented cell culture media (i.e. no HBOC) normalized O_2_ flux through the HF membrane for various 35:1 T-state PolyhHb and 30:1 R-state PolyhHb mixtures compared to hHb at Q = 40 mL/min and [PolyhHb]_total_ = [hHb] = 130 mg/mL. (B) Pure R-state normalized O_2_ consumption in the ECS versus PolyhHb T-state fraction, for various pO_2,in_s, Q = 40 mL/min and [PolyhHb] = 130 mg/mL.

Unsupplemented cell culture media was used as the control, while unmodified hHb was simulated for comparison. Unsupplemented cell culture media normalized O_2_ flux through the HF membrane for selected molar ratios of T- and R-state PolyhHb and hHb as a function of inlet pO_2_ is shown in Fig 8. For all HBOC molar ratios, the normalized O_2_ flux decreased as the inlet pO_2_ increased. At high pO_2,in_ (>80 mm Hg) the normalized O_2_ flux was similar to a 25% T-state PolyhHb fraction. At pO_2,in_ values ranging from 5–40 mm Hg, the simulated normalized flux of unmodified hHb was greater than all HBOC mixtures.

Pure R-state normalized O_2_ consumption by the hepatocytes housed in the ECS at various pO_2,in_s as a function of T-state PolyhHb fraction is shown in Fig 8. Here O_2_ consumption is used as an indicator of O_2_ delivery to the cultured hepatocytes. For low pO_2,in_s (<12 mm Hg), the rate of hepatocyte O_2_ consumption is greatest for pure R-state PolyhHb. At pO_2,in_ values close to 12 mmHg, the molar ratio of T-state to R-state PolyhHb has a negligible effect on O_2_ delivery. At increasing moderate pO_2,in_ values (12–40 mm Hg), O_2_ delivery increases with increasing molar ratio of T-state to R-state PolyhHb. At increasing high pO_2,in_ values (>40 mm Hg), the effect of the T-state to R-state PolyhHb mixture ratio on O_2_ consumption decreased.

**Fig 9:**
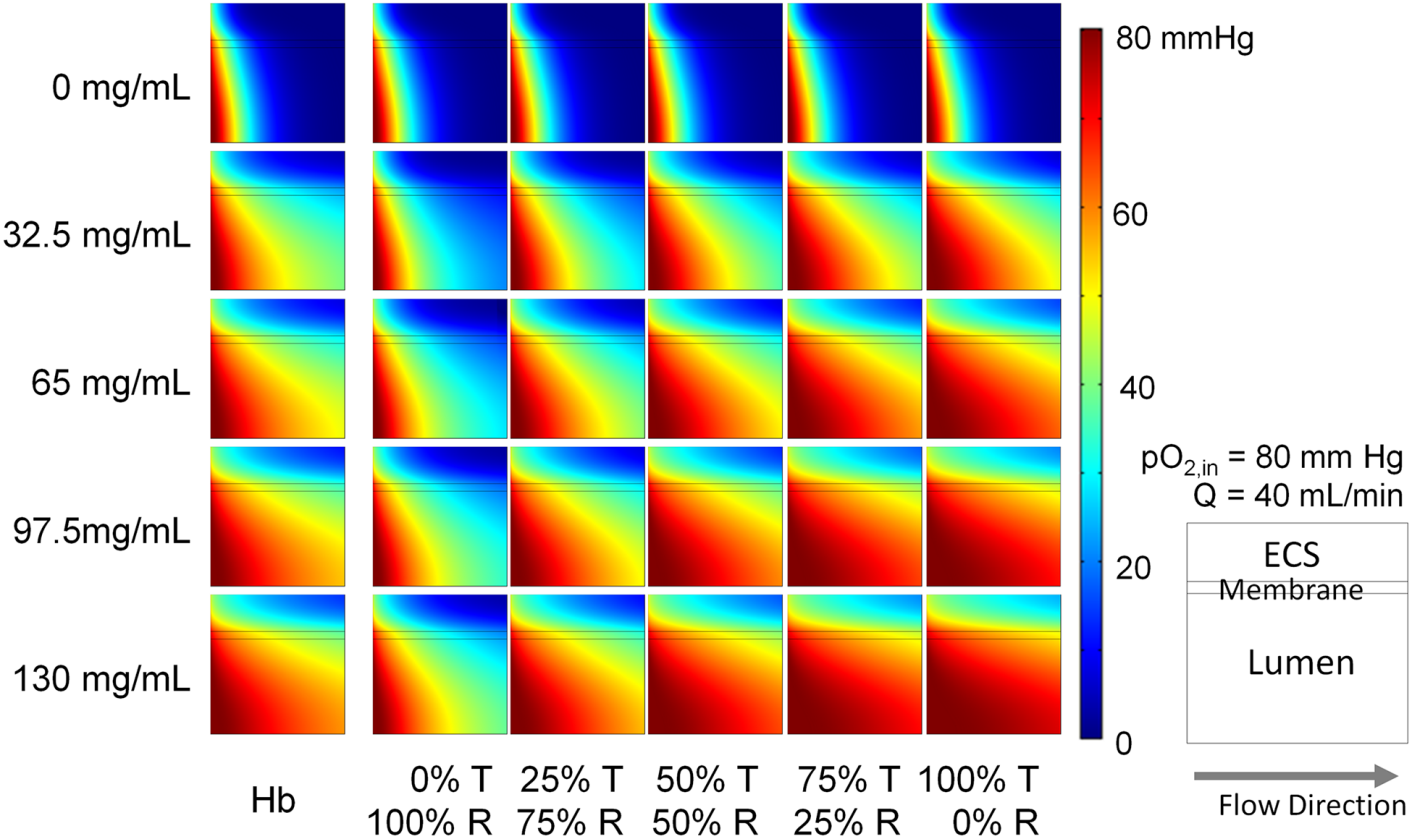
pO_2_ distribution in a single hollow fiber. Varying protein concentration was used for each species (y axis) for different hemoglobin species transfusion (x axis). The O_2_ content is depicted by a color scale from 0 to 80 mm Hg. Q = 40 mL/min pO_2,in_ = 80 mm Hg.

Simulated pO_2_ profiles for PolybHb mixtures and unmodified hHb supplemented cell culture media within the lumen, membrane, and ECS associated with a single HF are shown in Fig 9. The maximum protein concentration (130 mg/mL) was selected to approximate heme concentrations *in vivo* (i.e. in whole blood). Each frame in the figure represents a cross-sectional slice of a single HF unit. Flow in the system proceeds from left to right. The bottom of each panel corresponds to the HF centerline. In simulations with unsupplemented cell culture media, approximately 90% of the pO_2_ in the ECS was below 20 mm Hg. Oxygenation of the ECS improves with increasing protein concentration and increasing fraction of PolybHb in the T-state. These simulations demonstrate that the pO_2_ distributions for the 25% T-state PolybHb mixture are similar to that of unmodified hHb.

**Fig 10:**
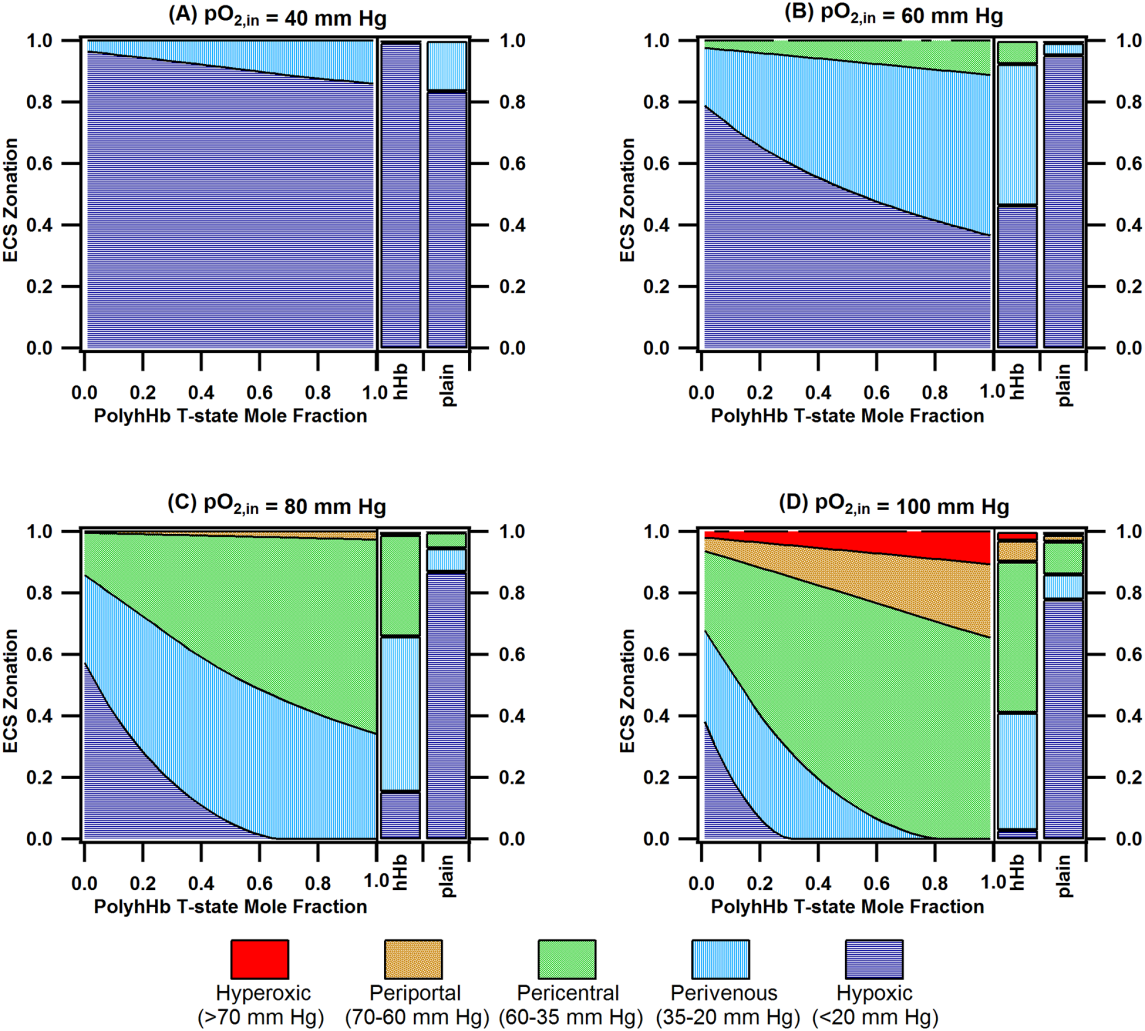
ECS zonation for PolyhHb fractions, hHb, and unsupplemented (plain) media for various pO_2,in_s. This figure displays the zonation (hyperoxic, periportal, perivenous, hypoxic) for PolyhHb fractions, hHb, and unsupplemented (plain) media where the pO_2,in_ is equal to (A) 40 mm Hg, (B) 60 mm Hg, (C) 80 mm Hg, and (D) 100 mm Hg. For this simulation Q = 40 mL/min and [PolyhHb]_total_ = [hHb] = 130 mg/mL.

Zonal heterogeneity in the liver sinusoid, which stems from O_2_ dependent regional variations in hepatocyte function, results in a “glucosat” in the liver [19,51]. This functionality is important in maintaining blood glucose levels during feeding and fasting periods. A variety of detoxification functions, which rely on sequential phase I and phase II metabolic enzymes, also requires proper zonation of these enzymes along the hepatic acinus [52]. Thus, replicating the zonation observed in the liver sinusoid is vital in bioartificial liver design. The ECS zonation plots for mixtures of PolybHb, unmodified hHb, and plain cell culture media at various pO_2,in_s are shown in Fig 10. Oxygenation zones within the ECS are classified as follows [51]: hypoxic (<20 mm Hg), perivenous (20–30 mm Hg), pericentral (35–60 mm Hg), periportal (60–70 mm Hg), and hyperoxic (>70 mm Hg). For unsupplemented cell culture media, a small fraction (12%-25%) of the hepatocytes are exposed to normoxic pO_2_ levels (20–70 mm Hg). For low pO_2,in_s (40 mm Hg and 60 mm Hg), the majority of the hepatocytes are exposed to hypoxic conditions (>40%) regardless of T-/R-state PolyhHb molar fraction. At pO_2,in_ = 80 mm Hg, the fraction of hepatocytes exposed to normoxic conditions with R-state PolyhHb (43%) is much less than the fraction of hepatocytes with T-state PolyhHb (99.9%). For pO_2,in_ at 80 mm Hg, the hypoxic region remains less than 5% for T-state PolyhHb fractions greater than 50%. The ratio of hepatocytes in the pericentral region to those in the perivenous region increases from 0.91 to 1.85 as the T-state PolyhHb fraction increases from 50% to 100%. For pO_2,in_ = 100 mm Hg, a fraction of the hepatocytes (4–7%) are exposed to hyperoxic conditions.

**Fig 11:**
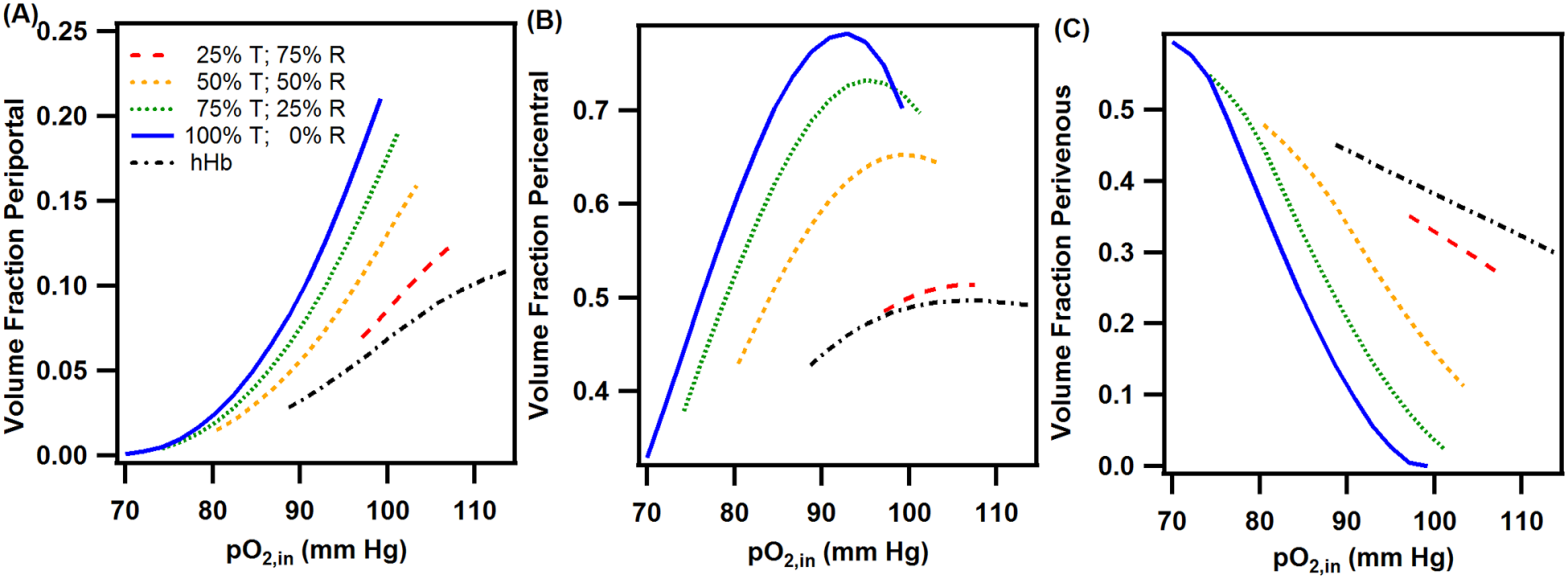
ECS zonation at varying pO_2,in_s for various PolyhHb mole fractions and pure hHb. This figure displays the zonation fractions of (A) periportal, (B) pericentral, and (C) perivenous regions for various PolyhHb mixtures and pure hHb where the sum of the hypoxic and hyperoxic fractions are less than 10% of the total volume. For this simulation Q = 40 mL/min and [PolyhHb]_total_ = [hHb] = 130 mg/mL.

At low pO_2,in_s (<40 mm Hg), unmodified hHb was able to deliver more O_2_ than the PolyhHbs synthesized in this study. This phenomenon likely results from the low cooperativity and high MW (i.e. lower diffusivity) of the synthesized PolyhHbs. For inlet pO_2_ ranges similar to the inlet conditions in the liver sinusoid (>60 mmHg), T-state PolyhHb delivered more O_2_ to the cells in the ECS. Furthermore, as the pO_2,in_ increased, the fraction of T-state PolyhHb required to outperform unmodified hHb decreased. This is likely due to the increased O_2_ dissociation rate constant of T-state PolyhHb. At low pO_2,in_s (<12 mmHg), the O_2_ delivery of R-state PolyhHb outperformed T-state PolyhHb. This can be explained by an increase in O_2_-offloading at low pO_2,in_s for R-state PolyhHb. This indicates that R-state PolyhHbs may be better suited to oxygenate hypoxic areas. To explore these effects further, we examined how the volume fraction of the periportal, pericentral, and perivenous sections varied as a function of the pO_2,in_ for PolyhHb mixtures and unmodified Hb. We then excluded any simulation results where the sum of the hypoxic and hyperoxic fractions was less than 10% of the total volume in the ECS. The results of this analysis are shown in Fig 11. Overall, pure T-state PolyhHb had the largest operating range where minimal hypoxic/hyperoxic behavior was observed (70–95 mm Hg). Increasing the mole fraction of R-state PolyhHb lead to increasingly narrow operating ranges. For pure R-state PolyhHb, no region was observed where the sum of the hypoxic and hyperoxic volume fractions were less than 10%. Decreasing the mole fraction of R-state PolyhHb in the PolyhHb mixture lead to broadened operational ranges. Interestingly, unmodified hHb had a similar operating curve to R-state PolyhHb. However, both the high mole fraction R-state PolyhHb mixtures and unmodified hHb solutions had less variation in the volume fractions for each zone compared to the high mole fraction T-state PolyhHb mixtures.

As expected, T-state PolyhHb has the potential to oxygenate a HF bioreactor better than R-state PolyhHb and unmodified hHb. These results are in agreement with the simulations performed by Zhou et al. [18]. The results of the finite element analysis indicate that O_2_ delivery can be controlled by adjusting the molar ratio of T-state to R-state PolyhHb in solution. When the T-state to R-state molar fraction drops below 50%, O_2_ delivery rapidly decreases. Therefore, it is recommended that mixtures of PolyhHb contain no less than 50% T-state PolyhHb. The percent of R-state PolyhHb may be tuned to both vary zonation or to increase O_2_ delivery to severely hypoxic regions. Finally, unmodified hHb may be favorable in maintaining relatively constant zonation if the pO_2,in_ varies. However, this provides much less flexibility in establishing different oxygenation zones due to the limited operating range of unmodified hHb. This is especially important considering the geometry of the hollow fiber bioreactor. *In vivo*, blood flows into the liver through both the portal and vein. This leads to an O_2_ gradient and functional zonation between arterioles and the central veins [19]. Replicating this oxygen gradient *in vitro* would necessitate a more complex bioreactor design. However, the results from the simulations indicate that application of the PolyhHb mixtures can vary the zonation despite not exhibiting the cooperative O_2_ binding behavior of native hHb.

## Conclusions

We have previously synthesized glutaraldehyde-cross-linked polymerized human Hb (PolyhHbs) with either low (T-state) or high (R-state) O_2_ affinity. In this study, we demonstrated that molar mixtures of T-state and R-state PolyhHbs can yield HBOCs with tunable O_2_ affinities. Additionally, O_2_ transport simulations performed in this study suggest that mixtures of PolyhHbs with T-state molar fractions greater than 50% are able to oxygenate a hepatic HF bioreactor better than those with T-state PolyhHb molar fractions less than 50%. Furthermore, by decreasing the T-state PolyhHb molar fraction, the ratio of pericentral to perivenous oxygenation was computationally calculated to decrease by 50% with minimal formation of hypoxic zones.

## Supporting information

S1 File In depth computational model methods and parameters. This file outlines the equations and parameters for the COMSOL model used to analyze oxygenation in a single hollow fiber contained in the bioreactor.
(DOCX)

S1 Table PolyhHb results. Table containing the biophysical properties for each of the PolyhHbs synthesized in this study.
(XLSX)
