# Supplementary material for: Mixtures of tense and relaxed state polymerized human hemoglobin regulate oxygen affinity and tissue construct oxygenation
Source: PLoS One. 2017 Oct 11;12(10):e0185988. doi: 10.1371/journal.pone.0185988 (PMC5636107; doi:10.1371/journal.pone.0185988)
Supplement: S1 File — This file outlines the equations and parameters for the COMSOL model used to analyze oxygenation in a single hollow fiber contained in the bioreactor. (DOCX) [file pone.0185988.s001.docx]

**Full Title: Mixtures of tense and relaxed state polymerized human hemoglobin regulate oxygen affinity and tissue construct oxygenation.**

Mixtures of polymerized hemoglobin facilitate tissue construct oxygenation.

Donald Andrew Belcher^1^, Uddyalok Banerjee^1^, Christopher Micheal Baehr^2^, Kristopher Emil Richardson^1^, Pedro Cabrales^3^, François Berthiaume^4^, Andre Francis Palmer^1*^

COMSOL Model Description

A finite element multiphysics analysis was performed in COMSOL Multiphysics to evaluate fluid and oxygen (O_2_) transport in a simulated hollow fiber (HF) bioreactor oxygenated with polymerized human hemoglobins (PolyhHbs). A schematic of the model with labeled boundaries is shown in Fig S1.


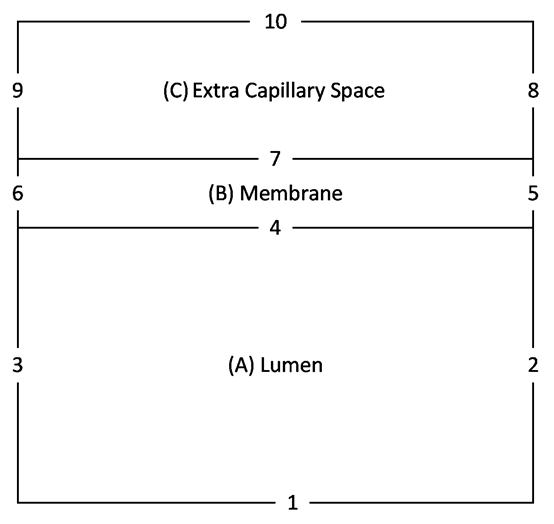


**Fig S1. COMSOL model layout.** The above figure shows the layout for the COMSOL model with the domains labeled with letters (A – C) and the boundaries labeled with numbers (1-10)

Partial differential equations describing momentum transport are used to evaluate the velocity profile in each of the subdomains. Flow in the lumen (A) is calculated using the Navier-Stokes equation (Eq. S1), while flow in the membrane (B) and the ECS (C) is calculated using Brinkman’s equation (Eq. S2).

$\rho\left( \bar{v}\cdot\bar{\nabla} \right)\bar{v}= \left[ -\bar{\nabla}P+\mu\nabla^{2}\bar{v} \right]$ Eq.S1

$\bar{v}=\frac{\kappa}{\mu}\bar{\nabla}\left( -P \right)+\kappa\nabla^{2}\bar{v}$ Eq.S2

Where ρ, v̅, μ, κ, and P represent the fluid density, fluid velocity, fluid viscosity, membrane intrinsic permeability, and pressure respectively. For the lumen, an axial symmetry condition is set at boundary 1. For the inlet (boundary 3) an average velocity is selected with an entry length of 1 cm. An outlet is assigned at boundary 2 setting the outlet pressure to 0 and suppressing all backflow. A second outlet at boundary 3 is assigned to the lumen membrane interface. At this boundary, the pressure is set to the pressure for the Brinkman domain boundary without suppressing backflow. For the Brinkman equation boundaries 5,6,8, and 9 are each defined as no slip. Boundary 10 is assigned as a symmetry condition. At the lumen membrane interface (4), the velocity field for the Brinkman flow is set to the boundary velocities for the Navier-Stokes flow in the lumen. The initial guesses for all flow parameters was set at 0.

Partial differential equations describing mass transport are used to evaluate transport of dissolved O_2_ and PolyhHb as shown in Equation S3.

$v\cdot\bar{\nabla}C=D\left[ \bar{\nabla}^{2}C \right]+R$ Eq.S3

Where C represents either the pO_2_, HBOC concentration, or hHb concentration. D represents the diffusivity of the respective species. R represents the rate of generation or depletion of the respective species. For O_2_ transport we used the fluid velocity field for the Navier-Stokes and Brinkman domains. O_2_ transport was performed with the species transport in porous media module. An axial symmetry condition was set at boundary 1. Boundaries 5, 6, 8, and 9 were each assigned as no-flux boundary conditions. Boundary 10 was assigned as a symmetry boundary condition. A concentration boundary condition was set at the inlet (boundary 3) for a given pO_2,in_. An outflow was assigned at boundary 2. In the membrane (B), the rate of O_2_ generation or depletion is zero. The rate of O_2_ consumption by the hepatocytes in the ECS (C) can be estimated by Michaelis-Menten kinetics (Eq. S4).

$R_{O_{2}}=-\frac{V_{M}pO_{2}}{k_{M}+pO_{2}}$ Eq.S4

Where V_M­_ is the maximum rate of O_2_ consumption, and k_M_ is the Michaelis-Menten coefficient. The rate of O_2_ dissociation from the HBOC occurs in the lumen (A) and is shown in Eq. S5 ([1](#_ENREF_1)).

$R_{HBOC-O_{2}}=k_{HBOC,off}\left[ HBOC_{total} \right]\left( S-\frac{Y_{e}}{1-Y_{e}}\left( 1-S \right) \right)$ Eq.S5

Where 𝑆 is the saturation (i.e. $[HBOC-O_{2}]/\left[ HBOC \right]_{total}$) and Y_e_ is the equilibrium HBOC saturation. Here the equilibrium HBOC saturation is estimated using the Hill Equation (Eq. 1) ([2](#_ENREF_2)). Initial guesses for the O_2_ distribution in all three domains are set to the inlet concentration.

Transport of the T-state and R-state PolyhHb are modelled in two separate physics entirely within the lumen (A). For both HBOCs, we used the transport of dilute species module. HBOC diffusivity was determined using the Stokes-Einstein relationship (Eq. S6).

$D_{HBOC}=\frac{k_{B}T}{6\pi\mu r_{HBOC}}$ Eq.S6

Where $k_{B}$, $T$, and $r_{HBOC}$ are the Boltzmann constant, temperature (37 °C), and average particle radius of the HBOC, respectively. Boundary 1 is assigned is set for axial symmetry. No flux conditions are assumed across the lumen-membrane interface (boundary 4). At the inlet (boundary 3), the concentration of both HBOC is set to the equilibrium saturation. An outflow condition is set at boundary 2. Conversion of oxygenated HBOC to deoxygenated HBOCs is performed according to Eq. S5.

Fluid flow was first evaluated using a stationary PARDISO solver with relative tolerance of 0.001 and automatic linearity. Velocity fields from this step of the simulation were then used to evaluate the mass transport of O_2_, T-state PolyhHb, and R-state PolyhHb using a MUMPS solver with a 0.001 relative tolerance. Inlet pO_2_, total HBOC concentration, HBOC fraction, and total flow rate were varied during simulations. **Table S1** lists the various physical constants and varied parameters used in the O_2_ transport model.

**Table S1.** Physical constants and varied parameters used in the O_2_ transport model.

| **Symbol** | **Simulation Parameter** | **Value** | **Units** | **Source** |
| --- | --- | --- | --- | --- |
| Physical Constants |  |  |  |  |
| $\boldsymbol{\alpha}$ | O_2_ solubility | 1.71 × 10^-3^ | mol/(m^3^∙mm Hg) | Sander ([3](#_ENREF_3)) |
| $\boldsymbol{\rho}_{\mathbf{0}}$ | Density of plain media | 1.0 | g/cm^3^ | Bird et al. ([4](#_ENREF_4)) |
| $\boldsymbol{\mu}_{\mathbf{0}}$ | Viscosity of plain media | 6.92 × 10^-4^ | kg/(m∙s) | Bird et al. ([4](#_ENREF_4)) |
| $\boldsymbol{\kappa}_{\boldsymbol{M}}$ | Membrane intrinsic permeability | 1.04 × 10^-20^ | m^2^ | Bear ([5](#_ENREF_5)) |
| $\boldsymbol{\epsilon}_{\boldsymbol{M}}$ | Membrane porosity | 0.8 |  | Bear ([5](#_ENREF_5)) |
| $\boldsymbol{\kappa}_{\boldsymbol{E}}$ | ECS intrinsic permeability | 1.4 × 10^-15^ | m^2^ | Nield and Bejan ([6](#_ENREF_6)) |
| $\boldsymbol{\epsilon}_{\boldsymbol{E}}$ | ECS porosity | 0.43 |  | Nield and Bejan ([6](#_ENREF_6)) |
| $\boldsymbol{D}_{\boldsymbol{O}_{\mathbf{2}},\boldsymbol{L}}$ | Diffusivity of O_2_ in the lumen | 3.0 × 10^-5^ | cm^2^/s | Foy et al. ([7](#_ENREF_7))  Piret and Cooney ([8](#_ENREF_8)) |
| $\boldsymbol{D}_{\boldsymbol{O}_{\mathbf{2}},\boldsymbol{M}}$ | Diffusivity of O_2_ in the membrane | 2.5 × 10^-5^ | cm^2^/s | Smith et al. ([9](#_ENREF_9)) |
| $\boldsymbol{D}_{\boldsymbol{O}_{\mathbf{2}},\boldsymbol{E}}$ | Diffusivity of O_2_ in the ECS | 2.0 × 10^-5^ | cm^2^/s | Hay et al. ([10](#_ENREF_10)) |
| $\boldsymbol{V}{}_{\boldsymbol{max}}$ | Maximum O_2_ consumption rate | 0.035 | mol/(m^3^∙s) | Smith et al. ([9](#_ENREF_9)) |
| $\boldsymbol{K}_{\boldsymbol{M}}$ | Michaelis constant | 3 | mm Hg | Hay et al. ([10](#_ENREF_10)) |
| $\boldsymbol{L}$ | Cartridge length | 12 | cm |  |
| $\boldsymbol{r}_{\boldsymbol{L}}$ | Radius of the lumen | 0.0100 | cm |  |
| $\boldsymbol{r}_{\boldsymbol{M}}$ | Membrane thickness | 0.0008 | cm |  |
| $\boldsymbol{r}_{\boldsymbol{K}}$ | ECS thickness | 0.0039 | cm |  |
| Varied Parameters |  |  |  |  |
| $\boldsymbol{p}_{\boldsymbol{O}\mathbf{2},\boldsymbol{in}}$ | Inlet O_2_ partial pressure | 0-140 | mm Hg |  |
| $\boldsymbol{Q}$ | Inlet flow rate | 1-50 | mL/min |  |
| $[\boldsymbol{HBOC}_{\boldsymbol{total}}]$ | Total HBOC concentration | 0-100% | 130 mg/mL |  |
| $\boldsymbol{\Phi}$ | R-state:T-state HBOC mole fraction | 0-1 |  |  |

# References

1. Chen G, Palmer AF. Hemoglobin-based oxygen carrier and convection enhanced oxygen transport in a hollow fiber bioreactor. Biotechnol Bioeng. 2009;102(6):1603-12.

2. Hill AV. The possible effects of the aggregation of the molecules of haemoglobin on its dissociation curves. J Physiol. 1910;40(4):iv-vii.

3. Sander R. Compilation of Henry’s Law Constants for Inorganic and Organic Species of Potential Importance in Environmental Chemistry [Available from:

<http://enviromap.utah.gov/businesses/E/EnSolutions/depleteduranium/performassess/compliancereport/docs/2014/07Jul/supinfo/appreferences/Sander1999.pdf>.

4. Bird RB SW, Lightfoot EN. Transport Phenomena, Revised 2nd Edition.: John Wiley & Sons, Inc.; 2006.

5. Bear J. Dynamics of Fluids in Porous Media. Trade Paperback Edition ed. New York: Dover Publications; 1988.

6. Nield DA BA. Convection in Porous Media.: Springer New York; 2013.

7. Foy BD, Rotem A, Toner M, Tompkins RG, Yarmush ML. A device to measure the oxygen uptake rate of attached cells: importance in bioartificial organ design. Cell Transplant. 1994;3(6):515-27.

8. Piret JM, Cooney CL. Model of oxygen transport limitations in hollow fiber bioreactors. Biotechnol Bioeng. 1991;37(1):80-92.

9. Smith MD, Smirthwaite AD, Cairns DE, Cousins RB, Gaylor JD. Techniques for measurement of oxygen consumption rates of hepatocytes during attachment and post-attachment. Int J Artif Organs. 1996;19(1):36-44.

10. Hay PD, Veitch AR, Smith MD, Cousins RB, Gaylor JD. Oxygen transfer in a diffusion-limited hollow fiber bioartificial liver. Artificial organs. 2000;24(4):278-88.
